# The Development of Acute Outreach Services in Aged Care Facilities (Nursing Homes): Using Telemedicine in Rural Areas

**DOI:** 10.1002/agm2.70013

**Published:** 2025-04-20

**Authors:** Daniel Kam Yin Chan

**Affiliations:** ^1^ University of New South Wales Sydney New South Wales Australia

## Abstract

Acute outreach services for older patients in nursing homes are being developed as our population ages rapidly, putting stress on the resources of hospitals and emergency departments. Since the COVID pandemic, the acceleration of the utilization of telemedicine has also been adopted for acute outreach services in metropolitan areas, with preliminary evidence supporting safety, but the generalization of the service to other settings, such as rural areas, is less clear.

The world is experiencing rapid aging according to a report from the United Nations. The demography will shift drastically as the fertility rate falls and people are living longer. In 2021, one in 10 people globally were aged 65 or above. In 2050, this age group is projected to rise to one in six people worldwide [1].

This demographic shift increases the demand for hospitalization and emergency department (ED) services [2]. In the United States, the older population accounts for over 20% of annual ED visits [3]. This also means an increase in the complexity of acute care cases, with older patients experiencing more geriatric syndromes such as delirium, cognitive impairment, and falls [4]. Furthermore, older people visiting EDs often present with multiple comorbidities, polypharmacy, complex physiologic changes, and multifaceted social and physical needs [4]. These demands have put extra stress on the healthcare system.

To address this growing need, acute outreach services to long‐term aged care facilities (or nursing homes) have been developed in recent years to help reduce hospitalizations and ED presentations from older people dwelling in these facilities [5, 6]. In Australia, these services have been found to be safe, accounting for no unexpected deaths and only 5.3% of older patients presenting to hospital for further investigation or treatment [5]. In one Australian study, ED presentation has been reduced by 10%, and during long‐term follow‐up, the hospitalization rate is reduced by 36% [5, 7]. Moreover, the cost‐benefit analysis shows that the ratio is 1:5, meaning that one dollar spent on acute outreach service will save five dollars if a patient is to be hospitalized. However, the caveat is that adherence to safe inclusion and exclusion criteria is essential, and the experience factor plays a paramount role [5, 7].

During the COVID pandemic, the use of telemedicine has been accelerated, including for nursing home patients. The efficacy and safety of its use for the treatment of acute illnesses other than COVID in the nursing home setting are less clear in the literature. The first paper of its kind revealed that the safety outcome is comparable to face‐to‐face [8]. Importantly, the condition under which this is carried out needs to be considered, as the study was undertaken by an experienced team and in a single‐center urban setting [8]. Furthermore, the use of telemedicine by geriatricians is accompanied by experienced frontline face‐to‐face nursing staff and is limited to the weekend service. Hence, the generalization to a rural setting, where recruitment of well‐trained nursing staff is more difficult, is unclear. The implication will be of immense importance if telemedicine for acute illnesses is found to be equally safe and efficacious in a rural setting compared with an urban situation because of its larger distance and relative lack of human resources such as geriatricians, making it an attractive innovation if feasible.

There are notable advantages of treating frail older people in nursing homes where they live. For instance, a more familiar environment in which they live may be associated with less occurrence of delirium compared with transfer to a new hospital environment. Less transfer to the hospital may also mean less pressure on the resources of the hospital and ED. However, there are also challenges. Older, frailer patients with multiple comorbidities carry a higher risk of deterioration and medical complications. More development of point‐of‐care investigations would be advantageous as well, particularly in rural settings. These may include point‐of‐care blood tests, ultrasound, and x‐ray. Treatment options may be limited in the nursing home as some drugs that require multiple injections or infusions daily are out of the question due to the distance factor. Experience in outreach service is invaluable, and the pathway of a matured service may require more caution and modification of existing urban inclusion and exclusion criteria to suit local rural needs. There is an additional technological challenge as Wi‐Fi services may not work as well, and software and hardware may not be as readily available as they are in urban areas.

The criteria for patient inclusion in these services must be carefully defined, as broad classifications like the Diagnostic Related Group (DRG) may overlook the nuances of individual patient needs. For instance, the risk of taking on a patient with the DRG of exacerbation of chronic obstructive pulmonary disease (COPD) may vary between individuals. The more severe cases (poorer oxygenation with rapid respiratory and heart rates) may be less suitable for telemedicine or acute outreach service, especially in a rural setting where the risk may be higher than an urban setting, the latter with more experienced staff and with easier transfer to hospital should the patient's condition deteriorate.

The journey of developing telemedicine for acute illnesses in the older population is still at its early stage, particularly in rural settings. Many challenges lie ahead and would require meticulous planning and collection of data to guide us as we move forward. Safety and quality of healthcare should not be compromised for the sake of efficiency. In other words, real efficiency or efficacy should embrace good safety and quality of care [9].

## Author Contributions

Daniel Kam Yin Chan opined, collated, and wrote the editorial.

## Conflicts of Interest

The author declares no conflicts of interest.
